# Genotype-Specific HPV mRNA Triage Improves Colposcopy Efficiency Compared with Cytology and ATHENA-Derived Triage: A Population-Based Study of HPV DNA-Positive Women

**DOI:** 10.3390/pathogens15060584

**Published:** 2026-05-28

**Authors:** Sveinung Wergeland Sørbye, Bente Marie Falang, Mona Antonsen, Elin Richardsen

**Affiliations:** 1 Department of Clinical Pathology, University Hospital of North Norway, 9006 Tromsø, Norway; mona.antonsen@unn.no (M.A.); elin.richardsen@unn.no (E.R.); 2 PreTect AS, 3490 Klokkarstua, Norway; bente.falang@pretect.no

**Keywords:** HPV screening, HPV DNA-positive women, HPV mRNA testing, E6/E7 oncogene expression, cervical intraepithelial neoplasia (CIN3+), molecular triage, ATHENA algorithm, colposcopy referral, cervical cancer prevention, genotype-specific risk stratification, liquid-based cytology

## Abstract

Background: Effective triage of HPV DNA-positive women is needed to reduce unnecessary colposcopies while maintaining cervical cancer prevention. We evaluated genotype-specific 7-type HPV E6/E7 mRNA triage in a real-world screening cohort. Methods: In this population-based single-centre study at the University Hospital of North Norway, 42,791 women underwent primary screening with the cobas HPV DNA assay during the period 2019–2024. Among 2370 HPV DNA-positive women, reflex cytology, 7-type HPV mRNA testing, and an ATHENA-derived triage strategy were compared using histologically confirmed CIN3+ through 31 December 2025 as the endpoint. Results: CIN3+ was detected in 60/2370 women (2.5%). Test positivity was 47.0% for cytology, 54.7% for ATHENA-derived triage, and 33.4% for HPV mRNA. Sensitivity was 78.3%, 86.7%, and 73.3%; specificity was 53.8%, 46.1%, and 67.7%; and PPV was 4.2%, 4.0%, and 5.6%, respectively. Colposcopies per CIN3+ detected were 23.7, 24.9, and 18.0. Conclusions: HPV mRNA triage improved referral precision and colposcopy efficiency, but with lower sensitivity than ATHENA-derived triage. These findings support 7-type HPV mRNA testing as a potentially useful molecular triage option where structured follow-up is feasible.

## 1. Introduction

The transition to primary HPV screening has changed the central challenge in cervical cancer prevention. In many organized screening programmes, cytology-based screening at shorter intervals has been replaced by HPV-based screening at longer intervals because HPV testing is more sensitive for cervical precancer [1]. Consequently, the key issue is no longer whether HPV infection can be detected, but how HPV-positive women should be managed: which women should be referred directly to colposcopy and biopsy, which can safely undergo repeat testing after 1 year, and which may return to longer screening intervals [2,3]. This question is particularly important because the risk associated with a positive HPV test is not constant, but depends on whether the infection is newly detected or reflects a prevalent or persistent infection. Large longitudinal data from a well-screened U.S. population have shown that CIN3+ rates are highest when HPV positivity reflects prevalent infection and lower when HPV detection is newly incident after prior negative tests [4,5].

This concept is closely aligned with modern risk-based management. Current American guidance is based on estimated CIN3+ risk derived from the current test result together with prior screening history, rather than on any single result in isolation [2,3]. Within such a framework, triage performance becomes crucial, because the triage test determines whether an HPV-positive woman is referred immediately, managed with surveillance, or returned to routine screening [2,3]. An important implication is that results from studies performed in previously untested populations may not be directly transferable to repeatedly screened populations. In women without prior HPV-based screening, a positive HPV result will include many longstanding, previously undetected infections, which tends to increase the positive predictive value for CIN3+. In contrast, among women with one or more prior negative HPV-based tests, the risk associated with a newly positive result is expected to be lower [4,5].

This issue is particularly relevant in our setting. At the Department of Clinical Pathology, University Hospital of North Norway, women attending cervical screening between 2016 and 2020 were routinely examined with cervical cytology together with a 3-type HPV mRNA test targeting HPV16, HPV18, and HPV45 [6]. Because this was a population-based cohort from a region where cervical cytology and 3-type HPV mRNA co-testing was routinely used during the period 2016–2020, a substantial proportion of women included in the present 2019–2024 cohort had likely undergone earlier HPV mRNA-based testing before their current HPV DNA-positive screening result. The cohort therefore does not represent a previously untested screening population, and the absolute CIN3+ risk after a positive HPV DNA result may consequently be lower than that reported in settings where women entered HPV-based screening without prior HPV testing history [4,5].

Against this background, triage markers that improve biological risk discrimination remain highly relevant. Cytology is still widely used for triage of HPV-positive women, but it is observer-dependent, only moderately reproducible, and provides limited information about whether an HPV infection is transient or transforming [7]. Partial HPV genotyping adds useful information by identifying women positive for HPV16 and HPV18, who on average have higher risk and may warrant more intensive management [8]. More recently, American enduring guidelines have further emphasized risk stratification among HPV-positive women by incorporating extended genotyping and differential management pathways for specific genotype groups [9]. However, genotype detection alone does not distinguish between the mere presence of viral DNA and transcriptionally active infection [10].

These considerations make HPV mRNA testing particularly relevant as a triage tool. By detecting E6/E7 oncogene expression, mRNA testing is directed at biologically active infections that are more closely linked to precancer progression than HPV DNA positivity alone [11]. A genotype-specific mRNA assay may therefore fit well within a risk-based management model, because it has the potential to identify a smaller subgroup of HPV-positive women at substantially higher short-term risk while sparing lower-risk women from immediate referral [4,11,12]. This may be especially important in populations with prior HPV-related testing history, where many newly detected HPV DNA-positive results may carry relatively low short-term risk [4].

Geographical variation in HPV genotype distribution is also relevant when evaluating genotype-specific triage assays. Large global and regional analyses have shown that HPV16 and HPV18 account for most invasive cervical cancers worldwide, while HPV31, HPV33, HPV45, HPV52, and HPV58 contribute a substantial additional fraction of cases, with HPV52 and HPV58 being particularly prominent in East Asian populations [13,14,15]. In a global causal attribution analysis including more than 110,000 women with invasive cervical cancer, HPV31/33/45/52/58 contributed an additional 15–20% of cases beyond HPV16/18 [13]. Similarly, meta-analyses and regional studies from East Asia have shown higher prevalence and attribution of HPV52 and HPV58 in cervical precancer and cancer than in many Western populations [14,15,16,17,18]. These regional differences are important because the clinical utility of any genotype-specific triage assay depends partly on the population-specific prevalence and carcinogenic contribution of the genotypes included in the assay.

The PreTect HPV-Proofer’7 assay detects E6/E7 mRNA from seven clinically important HPV types: 16, 18, 31, 33, 45, 52, and 58 [12]. These types account for a substantial proportion of cervical precancer and cancer worldwide, are included in the nonavalent HPV vaccine, and include genotypes with particular relevance in East Asian populations [13,14,15,19]. In contrast to pooled DNA positivity, genotype-specific mRNA testing may provide clinically relevant risk separation both across and within genotype groups by identifying infections with evidence of transcriptionally active oncogene expression [11,12]. The present study was designed to evaluate whether 7-type HPV mRNA testing can improve triage of HPV DNA-positive women in a real-world primary screening cohort. Specifically, we compared genotype-specific HPV mRNA triage with cytology and with an ATHENA-derived triage strategy, using histologically confirmed CIN3+ as the endpoint, and assessed whether mRNA results provide clinically meaningful risk stratification that could support more efficient colposcopy referral and follow-up decisions [20].

## 2. Materials and Methods

### 2.1. Study Design and Population

This study was conducted as a quality-assurance, population-based cohort investigation within the Norwegian Cervical Cancer Screening Programme. The work was performed at the Department of Clinical Pathology, University Hospital of North Norway (UNN), and represents an updated evaluation of HPV DNA-positive women screened between 1 January 2019 and 31 December 2024. Histological follow-up was available through 31 December 2025. The study design, inclusion criteria, and screening procedures have been described in detail previously [21].

The analysis was based on women attending routine primary cervical screening. In accordance with the screening programme, women with previous cervical cancer, recent treatment for CIN2+ or more severe disease, ongoing surveillance after abnormal screening results, or known immunosuppression were not part of the routine screening population. Women included in routine primary screening were not under active follow-up for unresolved previous abnormalities. In the Norwegian Cervical Cancer Screening Programme, women are eligible for routine primary screening only after previous abnormal findings have been resolved, whereas women with recent treatment for CIN2+ or more severe disease are managed in post-treatment surveillance rather than in routine primary screening. Thus, the cohort represents HPV DNA-positive women identified during routine screening, rather than women referred because of known persistent abnormalities or recent treatment history. Follow-up completeness was supported by the national call–recall system and by use of unique personal identification numbers, which allow linkage of screening, triage, biopsy, and treatment data across laboratories and clinical units in Norway.

The present study focused on women with a positive HPV DNA screening result who underwent reflex triage with liquid-based cytology and genotype-specific HPV mRNA testing. In addition, an ATHENA-derived triage strategy was evaluated analytically in the same cohort. The primary endpoint for the updated analysis was histologically confirmed CIN3+.

### 2.2. Primary HPV DNA Screening

Cervical samples were collected in PreservCyt^®^ medium (ThinPrep^®^, Hologic Inc., Marlborough, MA, USA) and analysed using the cobas^®^ 4800 HPV DNA assay (Roche Diagnostics, Mannheim, Germany). This assay provides separate results for HPV16 and HPV18 and a pooled positive result for the remaining included HPV types. Women with a positive HPV DNA result were eligible for triage analysis.

### 2.3. Triage Procedures

HPV DNA-positive samples were triaged by two laboratory-based methods: liquid-based cytology and the 7-type HPV E6/E7 mRNA assay PreTect HPV-Proofer’7 (PreTect AS, Klokkarstua, Norway). Cytology was classified according to routine diagnostic practice, and atypical squamous cells of undetermined significance or worse (≥ASC-US) was used as the threshold for a positive cytology triage result [22]. The mRNA assay detects E6/E7 transcripts from HPV types 16, 18, 31, 33, 45, 52, and 58. A positive result for any of these seven targets was considered a positive mRNA triage result.

To enable comparison with a genotype-based referral strategy, an ATHENA-derived triage strategy was also applied analytically to the same dataset [20]. This did not represent prospective implementation of the full ATHENA clinical management algorithm, but rather an approximation of selected ATHENA components based on the information available in the present cohort: cobas HPV16/18 genotyping and reflex cytology. Women positive for HPV16 and/or HPV18 DNA were classified as triage-positive irrespective of cytology, whereas women positive for other HPV DNA types were classified according to cytology, using ≥ASC-US as the threshold for triage positivity. Clinical follow-up during the study period followed the Norwegian Cervical Cancer Screening Programme and was not guided by the ATHENA-derived strategy.

### 2.4. Histological Follow-Up and Study Endpoint

Histological outcomes were identified through linkage to cervical biopsy and treatment records during follow-up through 31 December 2025. For the present analysis, CIN3+ was defined as CIN3, squamous cell carcinoma, adenocarcinoma in situ, or invasive adenocarcinoma. When more than one histological diagnosis was recorded for the same woman during follow-up, the most severe diagnosis was used in the endpoint analysis.

### 2.5. Statistical Analysis

The primary aim was to compare the clinical performance of cytology, an ATHENA-derived triage strategy, and 7-type HPV mRNA testing among HPV DNA-positive women, using histologically confirmed CIN3+ as the endpoint. The ATHENA-derived strategy was applied analytically and should be interpreted as an approximation based on available cobas HPV16/18 genotyping and cytology data, rather than as full implementation of the ATHENA clinical management algorithm. Test positivity, sensitivity, specificity, positive predictive value (PPV), and negative predictive value (NPV) were calculated for each triage strategy. Full 2 × 2 cross-tabulations, including true positives, false positives, true negatives, and false negatives, were generated for cytology, the ATHENA-derived strategy, and 7-type HPV mRNA triage.

Exact 95% confidence intervals (CIs) were calculated using the Clopper–Pearson method. Paired comparisons of triage positivity between strategies were performed using McNemar’s test, and *p*-values < 0.05 were considered statistically significant. Referral burden was evaluated as the proportion of women classified as triage-positive and as the number of colposcopies required to detect one case of CIN3+, assuming referral of all triage-positive women.

Additional analyses focused on genotype-specific risk stratification. Descriptive PPVs for CIN3+ were calculated for individual HPV mRNA genotypes, with denominators and CIN3+ case counts reported for each genotype. Because some women were positive for more than one mRNA genotype, genotype-specific counts were not mutually exclusive and were not summed across types. To avoid overinterpretation of sparse HPV18-specific data and asymmetrical comparison between pooled non-16/18 HPV DNA positivity and genotype-specific non-16/18 mRNA results, the main genotype-stratified analysis focused on HPV16/18 DNA-positive women according to corresponding HPV16/18 mRNA status. Combined cytology and HPV mRNA co-testing patterns were also examined to determine whether joint interpretation of the two triage tests provided additional discrimination of CIN3+ prevalence among HPV DNA-positive women.

All statistical analyses were performed using IBM SPSS Statistics for Windows, Version 29.0 (IBM Corp., Armonk, NY, USA, 2022).

### 2.6. Ethical Approval

The study was conducted in accordance with the Declaration of Helsinki and was approved by the Regional Committee for Medical and Health Research Ethics, North Norway (REK Nord; approval no. 203384; 18 December 2020). The project was evaluated as a quality-assurance study based on registry-linked and de-identified data. According to Norwegian regulations for this type of study, individual informed consent was not required.

## 3. Results

### 3.1. Screening Cohort and Analytic Triage Population

**Figure 1:**
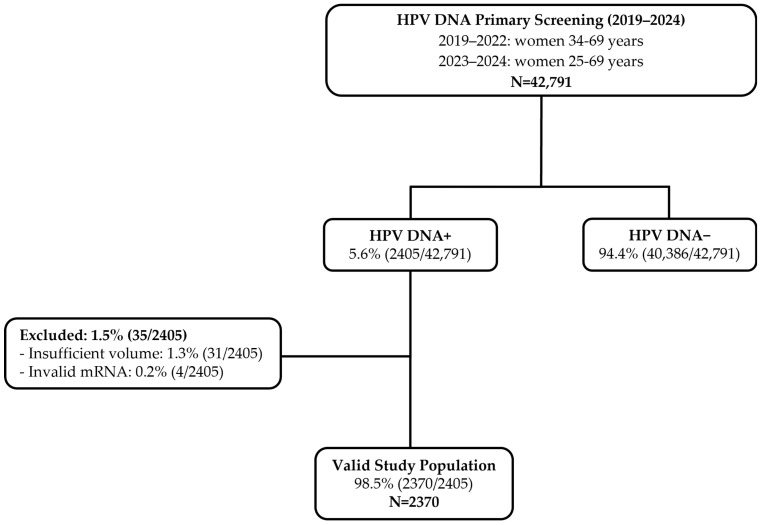
Flowchart of the study population. From January 2019 through December 2024, a total of 42,791 women underwent primary HPV DNA screening at the University Hospital of North Norway. Of these, 2405 women (5.6%) tested positive for HPV DNA and 40,386 (94.4%) tested negative. Among the HPV DNA-positive women, 35 were excluded because of insufficient sample volume (*n* = 31) or an invalid 7-type HPV mRNA test result (*n* = 4). The final analytic triage cohort therefore comprised 2370 HPV DNA-positive women with valid results available for reflex cytology, 7-type HPV mRNA testing, and follow-up outcome assessment. Histological follow-up was extended through December 2025. Abbreviations: HPV, human papillomavirus; DNA, deoxyribonucleic acid; mRNA, messenger ribonucleic acid; HPV DNA+, positive HPV DNA test result, i.e., HPV detected; HPV DNA−, negative HPV DNA test result, i.e., HPV not detected; N, number of women.

From January 2019 through December 2024, 42,791 women underwent primary HPV DNA screening at the University Hospital of North Norway. Among these, 2405 women (5.6%) tested positive for HPV DNA. After exclusion of 35 women because of insufficient sample volume (*n* = 31) or an invalid HPV mRNA test result (*n* = 4), 2370 women constituted the analytic triage cohort for the present study. All 2370 women had results available for reflex cytology, 7-type HPV mRNA testing, and follow-up outcome assessment in the updated dataset. Histological follow-up was extended through December 2025 (Figure 1).

### 3.2. Triage Positivity and Implications for Referral Burden

Within the cohort of 2370 HPV DNA-positive women, cytology was abnormal (≥ASC-US) in 1114 women (47.0%), whereas 1256 women (53.0%) had negative cytology. The 7-type HPV mRNA assay was positive in 791 women (33.4%) and negative in 1579 women (66.6%). Using the ATHENA-derived strategy, 1296 women (54.7%) would have been classified as triage-positive and 1074 (45.3%) as triage-negative. Thus, compared with cytology, the 7-type HPV mRNA test lowered triage positivity by 13.6 percentage points, whereas ATHENA increased triage positivity by 7.7 percentage points.

**Figure 2:**
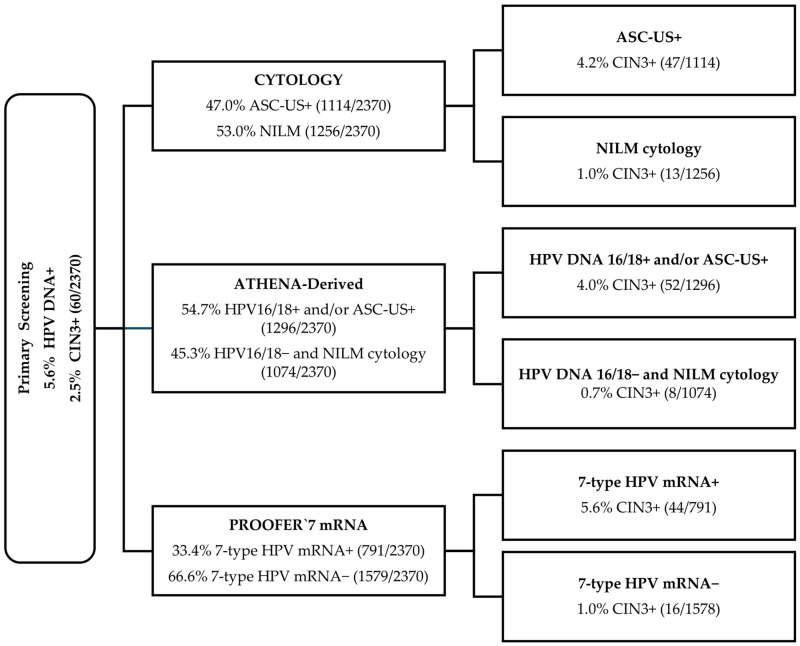
CIN3+ prevalence stratified by cytology, ATHENA-derived triage, and 7-type HPV mRNA triage. The figure shows observed CIN3+ prevalence among 2370 HPV DNA-positive women according to triage-positive and triage-negative results for each strategy. Overall CIN3+ prevalence was 2.5% (60/2370). CIN3+ prevalence was 4.2% in women with abnormal cytology, 4.0% in ATHENA-positive women, and 5.6% in HPV mRNA-positive women. Corresponding prevalences in triage-negative women were 1.0%, 0.7%, and 1.0%, respectively. Abbreviations: CIN3+, cervical intraepithelial neoplasia grade 3 or worse; HPV, human papillomavirus; DNA, deoxyribonucleic acid; mRNA, messenger ribonucleic acid; NILM, negative for intraepithelial lesion or malignancy; ASC-US, atypical squamous cells of undetermined significance; ASC-US+, cytological ASC-US or worse; HPV DNA 16/18+, detection of HPV type 16 and/or 18; HPV DNA 16/18−, neither HPV type 16 nor HPV type 18 detected; HPV mRNA+, positive 7-type HPV mRNA triage result; HPV mRNA−, negative 7-type HPV mRNA triage result; N, number of women.

The distribution of discordant results further illustrates the practical differences between the triage strategies. Under ATHENA, all 1114 women with abnormal cytology were triage-positive, and an additional 182 women with cytology-negative results would also have been referred because of HPV16/18 positivity. In contrast, comparison of cytology and 7-type HPV mRNA testing showed that 622 women with abnormal cytology were mRNA-negative, whereas 299 women with negative cytology were mRNA-positive. These findings indicate that ATHENA would expand referral volume beyond cytology alone, while 7-type HPV mRNA triage would concentrate referrals in a substantially smaller subset of HPV DNA-positive women (Figure 2).

### 3.3. CIN3+ Detection Rates and Risk Stratification by Triage Modality

During follow-up, 60 women were diagnosed with CIN3+, corresponding to an overall prevalence of 2.5% (60/2370) in the HPV DNA-positive triage cohort. Among women with abnormal cytology (≥ASC-US), 4.2% (47/1114) were diagnosed with CIN3+, compared with 1.0% (13/1256) among women with negative cytology. Using the ATHENA-derived strategy, 4.0% (52/1296) of triage-positive women had CIN3+, whereas only 0.7% (8/1074) of triage-negative women developed CIN3+. For the 7-type HPV mRNA assay, CIN3+ was identified in 5.6% (44/791) of mRNA-positive women, compared with 1.0% (16/1579) of mRNA-negative women (Figure 2). These findings show that all three triage approaches separated women into higher- and lower-risk groups, but the 7-type HPV mRNA test provided the strongest enrichment of CIN3+ among triage-positive women.

### 3.4. Comparative Diagnostic Accuracy of Cytology, ATHENA, and HPV mRNA Triage for CIN3+ Detection

The full 2 × 2 cross-tabulations for CIN3+ detection by each triage strategy are shown in Table 1. Cytology (≥ASC-US) identified 47 true-positive and 13 false-negative CIN3+ cases, whereas the ATHENA-derived strategy identified 52 true-positive and 8 false-negative cases. The 7-type HPV mRNA test identified 44 true-positive and 16 false-negative cases. Corresponding false-positive results were 1067 for cytology, 1244 for ATHENA, and 747 for 7-type HPV mRNA triage.

**Figure 3:**
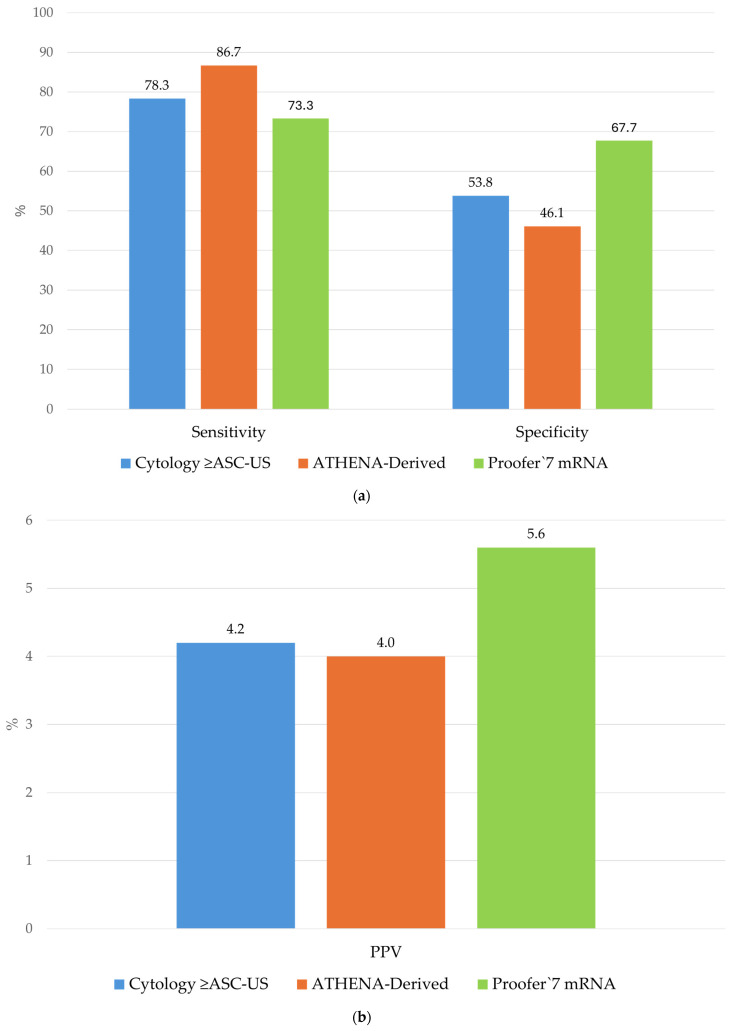
(**a**) Comparative sensitivity and specificity for CIN3+ detection by cytology, ATHENA-derived triage, and 7-type HPV mRNA triage. Sensitivity and specificity are shown for cytology (≥ASC-US), the ATHENA-derived triage algorithm, and the 7-type HPV E6/E7 mRNA assay among 2370 HPV DNA-positive women with histological follow-up through December 2025. ATHENA showed the highest sensitivity, whereas 7-type HPV mRNA triage showed the highest specificity. Abbreviations: CIN3+, cervical intraepithelial neoplasia grade 3 or worse; HPV, human papillomavirus; mRNA, messenger ribonucleic acid; E6/E7, HPV E6/E7 oncogene transcripts; ASC-US, atypical squamous cells of undetermined significance. (**b**) Comparative positive predictive value for CIN3+ detection by cytology, ATHENA-derived triage, and 7-type HPV mRNA triage. Positive predictive value (PPV) is shown for cytology (≥ASC-US), the ATHENA-derived triage algorithm, and the 7-type HPV E6/E7 mRNA assay among 2370 HPV DNA-positive women with histological follow-up through December 2025. PPV is shown separately because of the lower numerical range compared with sensitivity and specificity. The highest PPV was observed for 7-type HPV mRNA triage. Abbreviations: CIN3+, cervical intraepithelial neoplasia grade 3 or worse; HPV, human papillomavirus; mRNA, messenger ribonucleic acid; E6/E7, HPV E6/E7 oncogene transcripts; ASC-US, atypical squamous cells of undetermined significance; PPV, positive predictive value.

The derived diagnostic performance metrics are summarized in Table 2 and Figure 3a,b. ATHENA showed the highest sensitivity, 86.7% (95% CI: 75.4–94.1), followed by cytology at 78.3% (95% CI: 65.8–87.9) and the 7-type HPV mRNA test at 73.3% (95% CI: 60.3–83.9). In contrast, the 7-type HPV mRNA test achieved the highest specificity, 67.7% (95% CI: 65.8–69.6), compared with 53.8% (95% CI: 51.8–55.9) for cytology and 46.1% (95% CI: 44.1–48.2) for ATHENA (Figure 3a).

Positive predictive value was highest for the 7-type HPV mRNA test, 5.6% (95% CI: 4.1–7.4), whereas PPV was 4.2% (95% CI: 3.1–5.6) for cytology and 4.0% (95% CI: 3.0–5.2) for ATHENA (Figure 3b). PPV is shown separately because of the lower numerical range compared with sensitivity and specificity. Negative predictive value was high for all three strategies, ranging from 99.0% to 99.3%. Overall, ATHENA had the highest sensitivity, whereas 7-type HPV mRNA triage had the highest specificity and PPV.

### 3.5. Colposcopy Efficiency: Procedures Required per CIN3+ Detected

Assuming referral of all triage-positive women to colposcopy, cytology ≥ASC-US would result in 23.7 colposcopies per CIN3+ detected (1114/47), whereas the ATHENA-derived strategy would require 24.9 colposcopies per CIN3+ (1296/52). In comparison, the 7-type HPV mRNA strategy would require 18.0 colposcopies per CIN3+ detected (791/44; exact value 17.98), corresponding to a 24.1% reduction compared with cytology and a 27.8% reduction compared with ATHENA.

Paired comparisons of triage positivity confirmed significant differences between all strategies (McNemar’s test, *p* < 0.001 for all pairwise comparisons). Compared with cytology, 7-type HPV mRNA triage classified fewer women as triage-positive, whereas ATHENA classified more women as triage-positive.

### 3.6. Genotype-Specific Predictive Values

**Figure 4:**
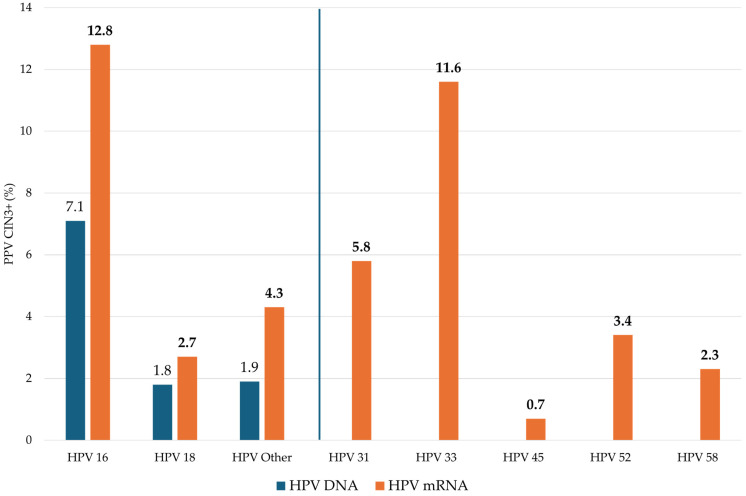
Genotype-specific CIN3+ prevalence according to HPV DNA and HPV mRNA results. Bars show observed CIN3+ prevalence for HPV16 DNA, HPV18 DNA, pooled non-16/18 HPV DNA, and genotype-specific HPV mRNA positivity. Estimates for less frequent genotypes should be interpreted cautiously because of small numbers of CIN3+ events. DNA-based and mRNA-based genotype categories are not fully symmetrical, because cobas provides partial DNA genotyping whereas Proofer’7 provides genotype-specific mRNA results for seven targeted types. Abbreviations: CIN3+, cervical intraepithelial neoplasia grade 3 or worse; HPV, human papillomavirus; DNA, deoxyribonucleic acid; mRNA, messenger ribonucleic acid.

Genotype-specific HPV mRNA results provided additional descriptive information on the distribution of CIN3+ within the HPV DNA-positive cohort (Table 3 and Figure 4). However, these estimates should be interpreted cautiously because several genotype-specific subgroups included few CIN3+ events, and because cobas HPV DNA testing and Proofer’7 mRNA testing do not provide directly symmetrical genotype information. The cobas assay reports HPV16 and HPV18 separately but groups the remaining HPV types as a pooled “other” category, whereas Proofer’7 provides genotype-specific mRNA results for seven targeted types.

Among individual mRNA genotypes, the highest point estimates for CIN3+ were observed for HPV16 mRNA (20/156; 12.8%) and HPV33 mRNA (8/69; 11.6%), followed by HPV31 mRNA (10/171; 5.8%), HPV52 mRNA (5/145; 3.4%), HPV18 mRNA (2/74; 2.7%), HPV58 mRNA (2/86; 2.3%), and HPV45 mRNA (1/153; 0.7%). These genotype-specific estimates should be regarded as descriptive point estimates rather than definitive genotype rankings, particularly for less frequent genotypes and subgroups with few CIN3+ events. Because some women had multiple mRNA-positive genotypes, genotype-specific counts are not mutually exclusive and should not be summed across types.

The overall PPV among mRNA-positive women was 5.6% (44/791). Taken together, these findings suggest that genotype-specific mRNA results may contribute to risk stratification within HPV DNA-positive women, but the precision of genotype-specific estimates is limited for several types, and comparisons with DNA-based partial genotyping should be interpreted with caution.

### 3.7. Refining Risk Stratification in HPV16/18 DNA-Positive Women

**Figure 5:**
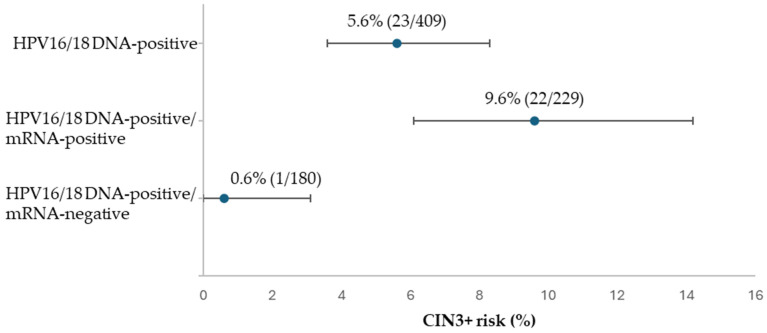
CIN3+ prevalence in HPV16/18 DNA-positive women according to corresponding HPV16/18 mRNA status. Points indicate observed CIN3+ prevalence, horizontal lines indicate 95% confidence intervals, and labels show percentages with numerators and denominators. Overall CIN3+ prevalence among HPV16/18 DNA-positive women was 5.6% (23/409; 95% CI: 3.6–8.3), increasing to 9.6% (22/229; 95% CI: 6.1–14.2) in women who were also HPV16/18 mRNA-positive and decreasing to 0.6% (1/180; 95% CI: 0.0–3.1) in women who were HPV16/18 mRNA-negative. Abbreviations: CIN3+, cervical intraepithelial neoplasia grade 3 or worse; HPV, human papillomavirus; DNA, deoxyribonucleic acid; mRNA, messenger ribonucleic acid; CI, confidence interval.

To avoid direct comparison between pooled non-16/18 HPV DNA positivity and genotype-specific non-16/18 mRNA results, genotype-stratified risk analysis was focused on HPV16/18 DNA-positive women. Among women positive for HPV16 and/or HPV18 DNA, the overall CIN3+ prevalence was 5.6% (23/409; 95% CI: 3.6–8.3). This increased to 9.6% (22/229; 95% CI: 6.1–14.2) in women who were also HPV16/18 mRNA-positive, but decreased to 0.6% (1/180; 95% CI: 0.0–3.1) in women who were HPV16/18 mRNA-negative (Figure 5).

These findings indicate that corresponding HPV16/18 mRNA positivity provides additional risk stratification within the HPV16/18 DNA-positive subgroup. The analysis also avoids overinterpretation of HPV18 alone, where the number of CIN3+ cases was low, and avoids an asymmetrical comparison between cobas pooled “other” HPV DNA positivity and genotype-specific mRNA results for HPV31, HPV33, HPV45, HPV52, and HPV58.

### 3.8. CIN3+ Prevalence According to Combined Cytology and HPV mRNA Triage

Additional stratification of observed CIN3+ prevalence was obtained when cytology and HPV mRNA results were evaluated jointly among HPV DNA-positive women (Table 4). The highest CIN3+ prevalence was observed in women with double-positive triage results, defined as ASC-US+ and HPV mRNA positivity, among whom 7.7% (38/492; 95% CI: 5.5–10.4) were diagnosed with CIN3+. In comparison, CIN3+ prevalence was 2.0% (6/299; 95% CI: 0.7–4.3) among women with normal cytology but positive HPV mRNA, and 1.4% (9/622; 95% CI: 0.7–2.7) among women with ASC-US+ but negative HPV mRNA. The lowest CIN3+ prevalence was observed in women with double-negative triage results, defined as normal cytology and negative HPV mRNA, among whom CIN3+ occurred in 0.7% (7/957; 95% CI: 0.3–1.5).

These findings show that combined cytology and HPV mRNA triage provided clinically meaningful stratification of observed CIN3+ prevalence within HPV DNA-positive women. In particular, HPV mRNA positivity identified a subgroup with higher observed CIN3+ prevalence, even among women with normal cytology, whereas women with double-negative results had very low observed CIN3+ prevalence.

## 4. Discussion

### 4.1. Principal Findings

The present study extends our earlier work [12,21] by showing that genotype-specific 7-type HPV E6/E7 mRNA testing remains a clinically useful triage approach for HPV DNA-positive women when evaluated against the more stringent endpoint CIN3+ and compared not only with cytology, but also with an ATHENA-derived strategy [20]. The main finding is that the evaluated strategies differed in their balance between immediate CIN3+ detection and referral burden. ATHENA achieved the highest sensitivity and detected the largest number of CIN3+ cases at the initial triage step, whereas Proofer’7 identified a smaller, more disease-enriched triage-positive subgroup with higher specificity, higher PPV, and fewer colposcopies per CIN3+ detected.

These findings illustrate the central clinical trade-off. ATHENA prioritizes immediate CIN3+ detection, but at the cost of substantially more referrals. In contrast, Proofer’7 improves referral precision and reduces colposcopy burden, but with lower sensitivity, meaning that some CIN3+ cases would not be selected for immediate colposcopy by mRNA triage alone. These cases should not be regarded as clinically irrelevant, but as cases requiring appropriate surveillance within the screening programme.

Overall, these results suggest that Proofer’7 functions primarily as a biologically informed risk-stratification tool. Its potential clinical value lies in improving referral precision by concentrating risk within a smaller subgroup of HPV DNA-positive women, while supporting surveillance of those at lower short-term risk.

### 4.2. Interpretation in Relation to Previous Studies

This overall pattern is consistent with our previous evaluations of the same assay [12,21]. In both the initial 2019–2021 implementation cohort and the expanded 2019–2023 analysis, 7-type HPV mRNA triage shifted referrals toward a smaller and more disease-enriched subgroup compared with cytology [12,21]. The present study confirms the same general pattern using the more stringent endpoint CIN3+ and with comparison against an ATHENA-derived strategy. Thus, the current findings extend our earlier work by showing that genotype-specific HPV mRNA testing continues to provide clinically relevant risk stratification when evaluated in a broader comparative triage framework.

At the same time, the absolute CIN3+ risks observed in the present cohort were lower than in our earlier CIN2+-based analyses [12,21], and this difference is biologically plausible. First, the endpoint in the current study was CIN3+ rather than CIN2+. Second, the study population was not HPV-screening-naïve. As outlined in the Introduction, many women in this cohort had undergone prior HPV-related testing, including earlier 3-type HPV mRNA testing at UNN [6]. Longitudinal data from other settings indicate that CIN3+ risk is lower when HPV positivity occurs after one or more preceding negative HPV tests than when the first known positive test represents a prevalent infection [4,5]. This context is important when interpreting the relatively low PPVs in the present study and when comparing these findings with results from populations entering HPV-based screening without prior HPV test history [4,5].

Our findings should also be interpreted in light of regional variation in HPV genotype distribution. The Proofer’7 assay targets HPV16, HPV18, HPV31, HPV33, HPV45, HPV52, and HPV58, which overlap with the oncogenic HPV types included in the nonavalent HPV vaccine. These genotypes are highly relevant in global cervical cancer prevention, but their relative contribution varies between populations. Large global and regional studies have shown that HPV16 remains the dominant genotype in invasive cervical cancer worldwide, while HPV52 and HPV58 are more frequent in East Asian populations than in many Western populations [13,14,15]. Chinese and Korean studies have likewise shown that HPV52 and HPV58 are common among HPV-positive women and in high-grade cervical lesions, although HPV16 remains the dominant genotype in invasive cancer [16,17,18,23]. This supports the biological and clinical relevance of genotype-specific risk stratification across regions, while also indicating that triage performance should be confirmed in populations with different genotype distributions. Further evaluation in geographically diverse screening settings, including East Asia, would therefore be valuable to assess the generalizability of the present findings.

### 4.3. Clinical Implications and Risk-Based Management

The findings are relevant in the context of contemporary risk-based management, in which clinical decisions increasingly depend on estimated CIN3+ risk and prior screening history rather than on any single test result in isolation [2,3]. From this perspective, the principal value of Proofer’7 is that it redistributes observed CIN3+ prevalence within the HPV DNA-positive group. The combined cytology/mRNA analysis was particularly informative: women with double-positive results had the highest observed CIN3+ prevalence, whereas double-negative women had very low observed CIN3+ prevalence. In addition, women with normal cytology but positive HPV mRNA had higher observed CIN3+ prevalence than women with ASC-US+ cytology but negative HPV mRNA. This suggests that evidence of transcriptionally active infection may provide clinically relevant information beyond low-grade morphological abnormalities alone in some HPV DNA-positive women [11].

The genotype-specific results further support the biological relevance of the assay [11]. Proofer’7 provided additional separation of observed CIN3+ prevalence within HPV DNA genotype strata, particularly within the combined HPV16/18 DNA-positive subgroup. However, genotype-specific estimates for less frequent types should be interpreted cautiously because some subgroups included few CIN3+ events and had wide confidence intervals. The relatively high point estimate observed for HPV33 mRNA positivity is biologically plausible, but should not be overinterpreted without confirmation in larger cohorts. Comparisons between cobas DNA genotyping and Proofer’7 mRNA genotyping are also not fully symmetrical, because cobas provides only partial DNA genotyping, whereas Proofer’7 provides genotype-specific mRNA results for seven targeted types.

The comparison with the ATHENA-derived strategy highlights the central management question: whether triage should prioritize maximal immediate CIN3+ detection or improved referral precision [20]. ATHENA favours sensitivity by referring HPV16/18-positive women directly to colposcopy and using cytology triage for women positive for other HPV types. Proofer’7 represents a different approach, favouring specificity and enrichment of CIN3+ among referred women. This strategy inevitably requires that HPV DNA-positive women not referred immediately are followed within a structured surveillance pathway. In repeatedly screened populations with relatively low absolute CIN3+ risk, the balance between immediate referral and safe surveillance may become increasingly important [4,5].

More broadly, these findings place genotype-specific HPV mRNA triage within the evolving landscape of cervical screening triage. Cytology remains widely used, but is dependent on morphological interpretation and has variable reproducibility [7]. Partial genotyping strategies such as ATHENA increase sensitivity by referring women with HPV16/18 DNA positivity directly to colposcopy, but they are based primarily on viral DNA presence rather than evidence of oncogenic activity [8,20]. Other biomarker-based triage approaches, including p16/Ki-67 dual staining and host-cell methylation markers, have also shown clinical value for risk stratification of HPV-positive women [24,25,26,27]. These methods provide different types of biological information: p16/Ki-67 dual staining reflects deregulated cell-cycle activity, methylation markers capture host-cell epigenetic changes associated with transformation, and HPV mRNA testing detects viral E6/E7 oncogene expression.

In this broader context, Proofer’7 may be regarded as a biologically distinct molecular triage approach whose potential contribution lies in combining genotype specificity with evidence of transcriptionally active infection [11,12]. This interpretation is supported by broader experience from genotype-specific HPV mRNA testing in routine practice [6]. Although the previous Norwegian co-testing study used a different assay configuration and clinical setting, the overall pattern was similar: mRNA positivity identified a smaller subgroup with higher observed CIN3+ prevalence, whereas double-negative women had very low observed proportions of CIN3+ and cervical cancer [6].

Taken together, these observations support the concept that genotype-specific HPV mRNA testing can provide biologically and clinically interpretable risk information beyond morphology alone [6,12,21]. Its most relevant role may depend not only on comparative clinical performance, but also on the screening context in which triage is applied. In particular, HPV mRNA testing may offer practical advantages in molecular triage workflows, including settings based on self-collected samples, where cytology- and dual stain-based triage are less directly applicable. Direct comparative studies with p16/Ki-67 dual staining and methylation-based markers would nevertheless be valuable to clarify how these approaches differ in performance, workflow suitability, and clinical utility across target populations with different screening histories, vaccination coverage, and acceptable trade-offs between sensitivity, specificity, referral burden, and safety of surveillance [2,3,11].

### 4.4. Strengths and Limitations

This study has several important strengths. It was conducted in a real-world, population-based screening setting, and screening, triage, histology, and treatment data could be linked through the national programme and unique personal identification numbers, ensuring near-complete follow-up. The cohort is substantially larger than in our earlier reports and includes extended histological follow-up through 31 December 2025. In addition, the study allowed direct comparison of three clinically relevant triage strategies—cytology, an ATHENA-derived strategy, and genotype-specific 7-type HPV mRNA testing—within the same HPV DNA-positive population and using the same underlying screening material. The genotype-specific mRNA assay also enabled more detailed biological stratification of observed CIN3+ prevalence than cytology alone or partial DNA genotyping.

The study also has limitations. First, it was observational and conducted at a single centre, which may limit generalisability to other screening settings and populations with different screening histories, HPV genotype distributions, vaccination coverage, screening intervals, and healthcare systems.

Second, HPV mRNA results did not prospectively guide clinical management; follow-up and referral were based on the approved screening algorithm using HPV DNA and cytology. Accordingly, some women who were cytology-negative but mRNA-positive were not referred immediately to colposcopy solely because of the mRNA result, and verification bias cannot be excluded. Some prevalent CIN3+ lesions in this subgroup may therefore have been detected later during follow-up rather than at the initial triage step. However, all HPV DNA-positive women remained within the follow-up pathway of the Norwegian Cervical Cancer Screening Programme, irrespective of cytology or mRNA status. Women with persistent HPV positivity are typically followed with repeat HPV-based testing at 12-month intervals, with referral to colposcopy and biopsy according to subsequent results. Histological follow-up was available through 31 December 2025, providing up to six years of follow-up for women screened in 2019. Thus, although verification bias cannot be excluded, the structured follow-up of all HPV DNA-positive women and the extended follow-up period reduce the likelihood that clinically relevant CIN3+ lesions were systematically missed.

Third, although Proofer’7 reduced referral burden and improved specificity and PPV, its sensitivity for CIN3+ was lower than that of the ATHENA-derived strategy. In the present cohort, ATHENA detected eight more CIN3+ cases than Proofer’7, but this was associated with 505 more colposcopy referrals overall. This illustrates the clinical trade-off between greater immediate sensitivity and reducing unnecessary referrals. CIN3+ cases not selected for immediate colposcopy by mRNA triage should therefore not be regarded as clinically irrelevant, but as cases requiring appropriate surveillance within the screening programme.

Fourth, the endpoint was CIN3+, which is clinically more stringent but less frequent than CIN2+, resulting in relatively few events and wider confidence intervals, particularly in genotype-specific subgroup analyses. No formal statistical power calculation was performed, because this was a population-based quality-assurance study including all eligible HPV DNA-positive women triaged with cytology and 7-type HPV mRNA testing during the study period. The relatively low number of CIN3+ events should therefore be considered when interpreting comparisons between triage strategies, particularly in subgroup analyses. To avoid overinterpretation of sparse data, HPV16 and HPV18 were combined in the revised genotype-stratified analysis, and estimates for individual mRNA genotypes were presented as descriptive point estimates with denominators rather than definitive genotype rankings.

Fifth, the study population was not HPV-screening-naïve. Women included in routine primary screening had no unresolved previous abnormal findings and were not in post-treatment surveillance after recent CIN2+ treatment. In addition, cytology with 3-type HPV mRNA co-testing was routinely used at the University Hospital of North Norway during the period 2016–2020, and many women included in the present 2019–2024 cohort may therefore have had prior negative HPV-related testing before the index HPV DNA-positive screening result [6]. This prior screening context may partly explain the relatively low CIN3+ prevalence and PPVs observed in the present study compared with populations entering HPV-based screening without previous HPV-related testing. However, detailed individual-level information on previous HPV positivity, genotype-specific persistence, and exact prior screening intervals was not included in the present analysis.

Sixth, individual HPV vaccination status was not available for analysis, and future genotype distributions and triage performance may change as vaccinated birth cohorts increasingly enter screening.

Finally, comparisons between the ATHENA-derived strategy and Proofer’7 should be interpreted with some caution. The ATHENA-derived strategy was based on partial HPV DNA genotyping (HPV16/18) combined with reflex cytology, whereas Proofer’7 was based on genotype-specific detection of E6/E7 mRNA from seven targeted HPV types. The observed differences therefore reflect not only alternative triage strategies, but also differences in biological target and assay design. The present study did not include a formal health-economic or cost-effectiveness analysis. Future studies should evaluate whether the reduction in colposcopy referrals observed with genotype-specific HPV mRNA triage translates into favourable cost-effectiveness in different screening settings.

In summary, the findings should be interpreted in light of the study’s single-centre design, the relatively low number of CIN3+ events, the prior screening history of the cohort, the lack of individual vaccination data, and the specific screening context in which the analyses were performed. These factors may limit direct extrapolation to other populations and future screening algorithms.

This balance may be particularly relevant in repeatedly screened populations, and potentially also in partially vaccinated cohorts, where residual disease is expected to become increasingly concentrated in a smaller subset of biologically important HPV infections. Future studies should clarify how Proofer’7 can best be integrated into contemporary management pathways, including whether its greatest value lies as a stand-alone molecular triage test or in combination with cytology in selected HPV DNA-positive subgroups.

## 5. Conclusions

In this single-centre, population-based cohort of HPV DNA-positive women, genotype-specific 7-type HPV E6/E7 mRNA triage showed higher specificity and PPV, lower triage positivity, and fewer colposcopies per CIN3+ detected than both cytology and the ATHENA-derived strategy. Although the ATHENA-derived strategy achieved higher sensitivity, it did so at the cost of substantially greater referral burden and lower specificity. Proofer’7 therefore appears to function as a more selective, risk-stratifying molecular triage approach that improves referral precision while reducing colposcopy use. Genotype-specific mRNA results and combined cytology/mRNA patterns provided additional clinically relevant risk stratification within HPV DNA-positive women, particularly in the HPV16/18 subgroup and in the contrast between double-positive and double-negative co-testing groups. Taken together, these findings support 7-type HPV mRNA testing as a clinically meaningful molecular triage option within contemporary risk-based cervical screening. Further independent studies should clarify how this strategy can best be integrated into screening algorithms and long-term follow-up pathways.

## Figures and Tables

**Table 1:** Full 2 × 2 cross-tabulations for CIN3+ detection by cytology, ATHENA-derived triage, and 7-type HPV mRNA triage among HPV DNA-positive women.

Triage Strategy	TP	FP	TN	FN
Cytology ≥ ASC-US	47	1067	1243	13
ATHENA-derived triage	52	1244	1066	8
7-type HPV mRNA	44	747	1563	16

Note: True positives (TP), false positives (FP), true negatives (TN), and false negatives (FN) are shown for each triage strategy using histologically confirmed CIN3+ as the endpoint. Abbreviations: ASC-US, atypical squamous cells of undetermined significance; CIN3+, cervical intraepithelial neoplasia grade 3 or worse; FN, false negative; FP, false positive; HPV, human papillomavirus; mRNA, messenger ribonucleic acid; TN, true negative; TP, true positive.

**Table 2:** Comparative diagnostic performance of cytology, ATHENA-derived triage, and P7 mRNA triage for CIN3+ detection.

Metric	Cyt ≥ ASC-US% (95% CI)	ATHENA% (95% CI)	P7 mRNA% (95% CI)
Sensitivity	78.3 (65.8–87.9)	86.7 (75.4–94.1)	73.3 (60.3–83.9)
Specificity	53.8 (51.8–55.9)	46.1 (44.1–48.2)	67.7 (65.8–69.6)
PPV	4.2 (3.1–5.6)	4.0 (3.0–5.2)	5.6 (4.1–7.4)
NPV	99.0 (98.2–99.4)	99.3 (98.5–99.7)	99.0 (98.4–99.4)

Sensitivity, specificity, positive predictive value (PPV), and negative predictive value (NPV) for histologically confirmed CIN3+ were calculated for cytology (≥ASC-US), the ATHENA-derived triage algorithm, and the 7-type HPV mRNA test. Confidence intervals (95% CI) are shown as exact estimates. Abbreviations: CIN3+, cervical intraepithelial neoplasia grade 3 or worse; ASC-US, atypical squamous cells of undetermined significance; CI, confidence interval; PPV, positive predictive value; NPV, negative predictive value; mRNA, messenger ribonucleic acid; P7, 7-type HPV E6/E7 mRNA assay PreTect HPV-Proofer’7.

**Table 3:** CIN3+ prevalence by HPV mRNA genotype among mRNA-positive women.

HPV mRNA Genotype	mRNA-Positive Women (*n*)	CIN3+ Cases (*n*)	Non-CIN3+ Cases (*n*)	PPV% (95% CI)
HPV 16 mRNA	156	20	136	12.8 (8.0–19.1)
HPV 18 mRNA	74	2	72	2.7 (0.3–9.4)
HPV 31 mRNA	171	10	161	5.8 (2.8–10.5)
HPV 33 mRNA	69	8	61	11.6 (5.1–21.6)
HPV 45 mRNA	153	1	152	0.7 (0.0–3.6)
HPV 52 mRNA	145	5	140	3.4 (1.1–7.9)
HPV 58 mRNA	86	2	84	2.3 (0.3–8.1)

Note: Genotype-specific counts are not mutually exclusive because some women were positive for more than one HPV mRNA genotype. PPV represents the observed proportion of CIN3+ among women positive for the specified mRNA genotype. Ninety-five percent confidence intervals (95% CI) were calculated using the exact binomial method (Clopper–Pearson). Abbreviations: CI, confidence interval; CIN3+, cervical intraepithelial neoplasia grade 3 or worse; HPV, human papillomavirus; mRNA, messenger ribonucleic acid; PPV, positive predictive value.

**Table 4:** CIN3+ prevalence according to combined cytology and HPV mRNA triage among HPV DNA-positive women.

Triage Group	*n*	CIN3+ Cases	CIN3+ Prevalence% (95% CI)
Double positive (ASC-US+ and HPV mRNA positive)	492	38	7.7 (5.5–10.4)
Normal cytology and HPV mRNA positive	299	6	2.0 (0.7–4.3)
ASC-US+ and HPV mRNA negative	622	9	1.4 (0.7–2.7)
Double negative (normal cytology and HPV mRNA negative)	957	7	0.7 (0.3–1.5)

Estimates represent the observed proportion of women with histologically confirmed CIN3+ during follow-up. Ninety-five percent confidence intervals (95% CI) were calculated using the exact binomial (Clopper–Pearson) method. Abbreviations: CIN3+, cervical intraepithelial neoplasia grade 3 or worse; HPV, human papillomavirus; DNA, deoxyribonucleic acid; mRNA, messenger ribonucleic acid; ASC-US, atypical squamous cells of undetermined significance; CI, confidence interval; n, number of women.

## Data Availability

The individual-level data presented in this study are not publicly available due to privacy and ethical restrictions under Norwegian data protection regulations. De-identified aggregate cross-tabulated data supporting the main analyses, including triage results and CIN3+ outcomes, may be made available from the corresponding author upon reasonable request.

## Abbreviations

The following abbreviations are used in this manuscript:

ASC-US+	Atypical squamous cells of undetermined significance or worse
CIN3+	Cervical intraepithelial neoplasia grade 3 or worse
E6/E7	HPV E6/E7 viral oncogenes
HPV	Human papillomavirus
LBC	Liquid-based cytology
mRNA	Messenger ribonucleic acid
NPV	Negative predictive value
P7	7-type HPV E6/E7 mRNA assay PreTect HPV-Proofer’7
PPV	Positive predictive value
SPSS	Statistical Package for the Social Sciences
UNN	University Hospital of North Norway
WHO	World Health Organization

## References

[1] Ronco G., Dillner J., Elfström K.M., Tunesi S., Snijders P.J.F., Arbyn M., Kitchener H., Segnan N., Gilham C., Giorgi-Rossi P. (2014). Efficacy of HPV-based screening for prevention of invasive cervical cancer: Follow-up of four European randomised controlled trials. Lancet.

[2] Perkins R.B., Guido R.S., Castle P.E., Chelmow D., Einstein M.H., Garcia F., Huh W.K., Kim J.J., Moscicki A.-B., Nayar R. (2020). 2019 ASCCP Risk-Based Management Consensus Guidelines for Abnormal Cervical Cancer Screening Tests and Cancer Precursors. J. Low. Genit. Tract Dis..

[3] Egemen D., Cheung L.C., Chen X., Demarco M., Perkins R.B., Kinney W., Poitras N., Lorey T., Castle P.E., Schiffman M. (2020). Risk Estimates Supporting the 2019 ASCCP Risk-Based Management Consensus Guidelines. J. Low. Genit. Tract Dis..

[4] Castle P.E., Kinney W.K., Xue X., Cheung L.C., Gage J.C., Poitras N.E., Lorey T.S., Katki H.A., Wentzensen N., Schiffman M. (2019). Role of Screening History in Clinical Meaning and Optimal Management of Positive Cervical Screening Results. J. Natl. Cancer Inst..

[5] Hammer A., Demarco M., Campos N., Castle P., Wentzensen N., Gravitt P., Befano B., Poitras N., Lorey T., Kinney W. (2020). A Study of the Risks of CIN3+ Detection after Multiple Rounds of HPV Testing: Results of the 15-Year Cervical Cancer Screening Experience at Kaiser Permanente Northern California. Int. J. Cancer.

[6] Sørbye S.W., Falang B.M., Antonsen M., Richardsen E. (2026). Cervical Cytology and HPV16/18/45 mRNA Co-Testing Improve Risk Stratification in Routine Clinical Practice. Cancers.

[7] Luttmer R., Dijkstra M.G., Snijders P.J.F., Berkhof J., van Kemenade F.J., Rozendaal L., Helmerhorst T.J., Verheijen R.H., Ter Harmsel W.A., van Baal W.M. (2016). p16/Ki-67 dual-stained cytology for detecting cervical (pre)cancer in high-risk HPV-positive women. Mod. Pathol..

[8] Demarco M., Egemen D., Raine-Bennett T.R., Cheung L.C., Befano B., Poitras N.E., Lorey T.S., Chen X., Gage J.C., Castle P.E. (2020). A Study of Partial Human Papillomavirus Genotyping in Support of the 2019 ASCCP Risk-Based Management Consensus Guidelines. J. Low. Genit. Tract Dis..

[9] Massad L.S., Clarke M.A., Perkins R.B., Garcia F., Chelmow D., Cheung L.C., Darragh T.M., Egemen D., Lorey T.S., Nayar R. (2025). Applying Results of Extended Genotyping to Management of Positive Cervicovaginal Human Papillomavirus Test Results: Enduring Guidelines. J. Low. Genit. Tract Dis..

[10] Falcinelli C., Claas E., Kleter B., Quint W. (1992). Detection of the human papilloma virus type 16 mRNA-transcripts in cytological abnormal scrapings. J. Med. Virol..

[11] Origoni M., Cristoforoni P., Carminati G., Stefani C., Costa S., Sandri M.T., Mariani L., Preti M. (2015). E6/E7 mRNA Testing for Human Papilloma Virus-Induced High-Grade Cervical Intraepithelial Disease (CIN2/CIN3): A Promising Perspective. Ecancermedicalscience.

[12] Sørbye S., Falang B.M., Antonsen M., Mortensen E. (2025). Genotype-Specific HPV mRNA Triage Improves CIN2+ Detection Efficiency Compared to Cytology: A Population-Based Study of HPV DNA-Positive Women. Pathogens.

[13] Wei F., Georges D., Man I., Baussano I., Clifford G.M. (2024). Causal attribution of human papillomavirus genotypes to invasive cervical cancer worldwide: A systematic analysis of the global literature. Lancet.

[14] Guan P., Howell-Jones R., Li N., Bruni L., de Sanjosé S., Franceschi S., Clifford G.M. (2012). Human papillomavirus types in 115,789 HPV-positive women: A meta-analysis from cervical infection to cancer. Int. J. Cancer.

[15] Chan P.K.S., Ho W.C.S., Chan M.C.W., Wong M.C.S., Yeung A.C.M., Chor J.S.Y., Hui M. (2014). Meta-analysis on prevalence and attribution of human papillomavirus types 52 and 58 in cervical neoplasia worldwide. PLoS ONE.

[16] Chen W., Zhang X., Molijn A., Jenkins D., Shi J.F., Quint W., Schmidt J.E., Wang P., Liu Y.L., Li L.K. (2009). Human papillomavirus type-distribution in cervical cancer in China: The importance of HPV 16 and 18. Cancer Causes Control.

[17] Lee E.H., Um T.H., Chi H.S., Hong Y.J., Cha Y.J. (2012). Prevalence and distribution of human papillomavirus infection in Korean women as determined by restriction fragment mass polymorphism assay. J. Korean Med. Sci..

[18] So K.A., Lee I.H., Lee K.H., Hong S.R., Kim Y.J., Seo H.H., Kim T.J. (2019). Human papillomavirus genotype-specific risk in cervical carcinogenesis. J. Gynecol. Oncol..

[19] de Sanjosé S., Serrano B., Tous S., Alejo M., Lloveras B., Quirós B., Clavero O., Vidal A., Ferrándiz-Pulido C., Pavón M.A. (2019). Burden of Human Papillomavirus (HPV)-Related Cancers Attributable to HPVs 6/11/16/18/31/33/45/52 and 58. JNCI Cancer Spectr..

[20] Castle P.E., Stoler M.H., Wright T.C., Sharma A., Wright T.L., Behrens C.M. (2011). Performance of carcinogenic human papillomavirus (HPV) testing and HPV16 or HPV18 genotyping for cervical cancer screening of women aged 25 years and older: A subanalysis of the ATHENA study. Lancet Oncol..

[21] Sørbye S.W., Falang B.M., Antonsen M. (2023). Performance of a 7-Type HPV mRNA Test in Triage of HPV DNA Primary Screen Positive Women Compared to Liquid-Based Cytology. J. Mol. Pathol..

[22] Nayar R., Wilbur D.C. (2017). The Bethesda System for Reporting Cervical Cytology: A Historical Perspective. Acta Cytol..

[23] Oh J.K., Alemany L., Suh J.I., Rha S.H., Muñoz N., Bosch F.X., Quint W., Lloveras B., Klaustermeier J.E., de Sanjosé S. (2010). Type-specific human papillomavirus distribution in invasive cervical cancer in Korea, 1958–2004. Asian Pac. J. Cancer Prev..

[24] Clarke M.A., Wentzensen N., Perkins R.B., Guido R.S., Schiffman M., Chelmow D., Einstein M.H., Garcia F., Huh W.K., Kim J.J. (2024). Recommendations for Use of p16/Ki-67 Dual Stain for Management of Individuals Testing Positive for Human Papillomavirus. J. Low. Genit. Tract Dis..

[25] Wentzensen N., Clarke M.A., Bremer R., Poitras N., Tokugawa D., Goldhoff P.E., Castle P.E., Schiffman M., Kingery J.D., Grewal K.K. (2019). Clinical Evaluation of Human Papillomavirus Screening with p16/Ki-67 Dual Stain Triage in a Large Organized Cervical Cancer Screening Program. JAMA Intern. Med..

[26] Bonde J., Floore A., Ejegod D., Vink F.J., Hesselink A., van de Ven P.M., Straume O., Pedersen H., Cuschieri K., Bevilacqua F. (2021). Methylation Markers FAM19A4 and miR124-2 as Triage Strategy for Human Papillomavirus-Positive Women: A Large European Multicenter Study. Int. J. Cancer.

[27] Schreiberhuber L., Barrett J.E., Wang J., Friman H., Dillner L., Lagheden C., Naeem A., Lei J., Pils S., Czene K. (2024). Cervical Cancer Screening Using DNA Methylation Triage in a Real-World Population. Nat. Med..

